# Some considerations on the use of space sound absorbers with next-generation materials reflecting COVID situations in Japan: additional sound absorption for post-pandemic challenges in indoor acoustic environments

**DOI:** 10.14324/111.444/ucloe.000012

**Published:** 2020-11-18

**Authors:** Kimihiro Sakagami, Takeshi Okuzono

**Affiliations:** 1 Environmental Acoustic Laboratory, Department of Architecture, Graduate School of Engineering, Kobe University, Rokko, Nada, Kobe 657-8501, Japan

**Keywords:** built environment, sound absorption, microperforated panel, permeable membrane, post-pandemic ‘new style’, indoor acoustic environment

## Abstract

In this study, we first point out the possible acoustic problems associated with the post-pandemic operation of built environments. In particular, we focus on the problem of acoustic deficiency due to the lack of absorption. This deficiency, which is likely to be encountered in most enclosed spaces in a range of establishments, is due to the reduced number of audience members or users of the space as a result of social distancing. As one of the promising solutions to this problem, we introduce a sound absorption technique using three-dimensional (3D) space sound absorbers developed through our recent research projects. Significantly, the type of sound absorber proposed herein is made of materials that are especially suited to hygiene considerations. The materials are microperforated panels (MPPs) and permeable membranes (PMs), both of which are easily washable and sanitised. Furthermore, we point out that 3D-MPP or PM space absorbers possess the additional value of aesthetic designability.

## Introduction

### Background: sound absorption and the ‘new lifestyle’

Among the built environments in cities, there are various types of public spaces used for different purposes. For example, in railway stations, airports, schools and meeting rooms in community halls, acoustical problems are often encountered due to insufficient sound absorption. This occurs not only in public spaces but also in private spaces, such as dwellings, meeting rooms and offices in commercial premises. These spaces also often suffer from excessive reverberations, caused by insufficient sound absorption, resulting in various acoustical deficiencies, for example, lower speech intelligibility, higher noise levels, the feeling of ‘noisiness’, etc.

In order to avoid these problems, additional sound absorption treatment is the only effective method of passive treatment. However, sound absorption treatments are usually made on the interior walls of rooms, which leads to a change in the design of the interior surfaces. Furthermore, conventional porous and fibrous materials for sound absorption are not suitable for interior surfaces unless appropriate facings are applied. This can also cause a problem due to insufficient strength or textural design of the interior surfaces; thus, in many cases, it may be desirable to avoid these methods.

In the current situation, following the COVID-19 outbreak, most people’s activities are conducted according to a set of suggested guidelines known as the ‘new style’, ‘new lifestyle’ or ‘new normal’ in each country. For example, in Japan, several new guidelines for the conduct of everyday activities have been proposed [1]. According to these guidelines, people must maintain a distance from other people, and this results in limitation of the use of public spaces. For example, the Ministry of Health and Labour in Japan suggests that all activities involving gatherings should be held in a space with the capacity for a group twice as large as the number of people actually gathering. Guidelines for various types of events and activities have been published [2]. In practice, this is applied not only to live performances or events in large auditoriums but also to small performance spaces and non-performance spaces such as meeting rooms. (See also Article Note 1.)

**Figure 1:**
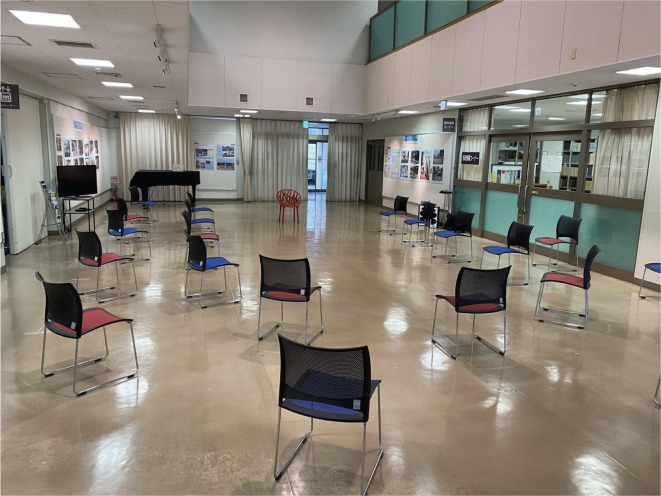
An example of a setting for a performing arts gathering in a multi-purpose room in a municipal hall. Before the pandemic, the room accommodated 60 audience members, but capacity is limited after the COVID-19 outbreak. One can see only 20 seats for the audience members, in order to maintain social distancing (photo courtesy of T. Soki).

As an example of this ‘new style’ of operation of smaller multi-purpose spaces, Fig. 1 shows a seating arrangement in a multi-purpose room in a municipal hall, where concerts and performing arts gatherings are regularly held, in a certain city in Japan. In this case, the room can usually hold 60 audience members, but after the COVID-19 outbreak, the number of audience members is limited to 20. This drastic reduction of the audience is likely to affect the acoustics of this room.

The reduction in audience numbers can result in lower sound absorption within the space, which leads to longer reverberation, lower speech intelligibility, etc., in events requiring verbal communication, such as meetings, symposia or theatrical performances. Such a situation, in many cases, would not have occurred without the COVID-19 pandemic: for example, in performances without an audience for broadcasting programmes, one may hear reverberant sounds, which would not be the case without the reduced numbers due to the pandemic. The problem of lower speech intelligibility can become more critical with larger distances between speakers and listeners. Moreover, face shields or mouth masks, which people are advised to wear during conversation, are often used. It has been reported that masks affect the acoustic characteristics of voices drastically [3]. According to the results [3], the effect of a face mask appears at 1 kHz and above as a reduction of c. 3–7 dB, which is less than that of a face shield, which is more drastic (3–12 dB): the authors consider that both effects are regarded as large. These results indicate that face shields and masks reduce the high frequency components of voices, which affects the loudness and timbre, and eventually causes a deterioration in the sound. This point is clearly shown in their demonstration recordings available in [3]. Not only is there an effect on speech intelligibility, but also on the sound quality of musical performances. Although in music this may be a matter of subjective preference for performers and listeners, it becomes more than a matter of individual preference when the intelligibility of speech is affected. According to the authors’ questionnaire survey (performed in June 2020, unpublished) of university students, some students pointed out that face masks cause a decline in speech intelligibility and speech communication quality. As most people have experienced, it is rather difficult to speak clearly with a face mask, which also causes another effect on speech intelligibility. Therefore, regarding speech intelligibility, it is already deteriorated in many cases before it is affected by room acoustic characteristics. In this respect, improving the room acoustic condition can save the further deterioration of speech communication ability.

### Sound absorption technology

Sound absorption technique is practically the only passive solution to controlling the acoustics of an enclosed sound field [4]. Therefore, it has been studied extensively [5]. There are three main types of sound absorbers: (1) porous and fibrous type absorbers, (2) panel- or membrane-type resonant absorbers and (3) Helmholtz resonator-type absorbers (including perforated panels).

All these methods can obviously contribute to the improvement of the mentioned problem of lower absorption. However, nowadays we must consider which type of absorber is the best from a hygiene point of view. Considering the nature of porous and fibrous materials, they are obviously not suitable in this regard. Washable or easily sanitised materials are more advantageous for post-pandemic applications. Therefore, sheet- or panel-like materials are advantageous, as they can be cleaned by applying (e.g. spraying) disinfectants relatively easily. Therefore, panel- or membrane-type resonant absorbers, or perforated (including microperforated) panels are likely to be good choices.

There are recently developed materials such as metal wool or porous metal, which can be more advantageous than traditional porous/fibrous material in this regard; however, in this commentary we focus on the panel or membrane materials, which are in many cases lightweight and flexible.

However, panel-like materials are usually used with a rigid back wall and an air cavity placed between the absorber and the wall. This requires a change in the interior surfaces of buildings. In many cases it is difficult to make a permanent change of the interior surface to accommodate sound absorption materials, as it can require substantial renovation of the building. In such a case, space sound absorbers, which can be placed mainly on floors are one of the alternative methods used to control the acoustics of the room, to reduce excessive reverberations. This is an effective alternative of flexible and reversible design not only for temporary improvements for the post-pandemic ‘new style’ but also in multipurpose spaces, etc., in ‘usual’ situations. Another way of using these absorbers is to suspend them from the ceiling, which is widely known; however, this usage is likely to require more constructive cost and effort than floor-mounted types. In the following, some basic ideas about space sound absorbers with panel- or sheet-like materials are presented.

### Alternative sound absorption materials and structures suitable for post-pandemic applications

As already mentioned, porous and fibrous materials raise difficulties in their application for the purposes discussed in the present study, whereas membrane and perforated panels are promising alternatives. There are two types of membrane material: impermeable, which does not permit airflow and reflects sound itself, and permeable, which uses acoustic flow resistance. Considering the use of a membrane for a space absorber, a permeable membrane (PM) is a better and more advantageous choice. Regarding perforated panels, conventional and traditional perforated panels with larger holes (larger than a few millimetres) are not suitable because of their lower absorptivity. Therefore, the most promising alternatives among these are microperforated panels, which are thin flexible panels with submillimetre holes, below a 1% perforation ratio. They are usually used on interior surfaces; however, they can also be used as free-standing space sound absorbers.

Recently, we proposed three-dimensional microperforated space sound absorbers (3D-MPAs) of various types: cylindrical, rectangular and spherical [6–8]. A similar concept has also been applied to PMs, and we have proposed 3D PM space sound absorbers (3D-PMAs) [9,10]. In the following, the main results of our projects on the 3D-MPA and 3D-PMA designs are introduced. All the absorbers introduced in this commentary can be either put on floors or suspended from ceilings, are made of light-weight plastics or fabrics that are washable or easily sanitised, and are potentially designable, which may be one of solutions for improving the acoustics of built environments for use in the ‘new style’. Here, we consider mainly public spaces such as auditoria, meeting rooms, etc., however, the idea is also applicable in a residential building: in that case, considering the scale of the space, we would suggest to use it with some additional value, for example, a lampshade, etc., for everyday use, which is mentioned later.

## Space sound absorbers with next-generation materials

### Space sound absorbers with microperforated panels

**Figure 2:**
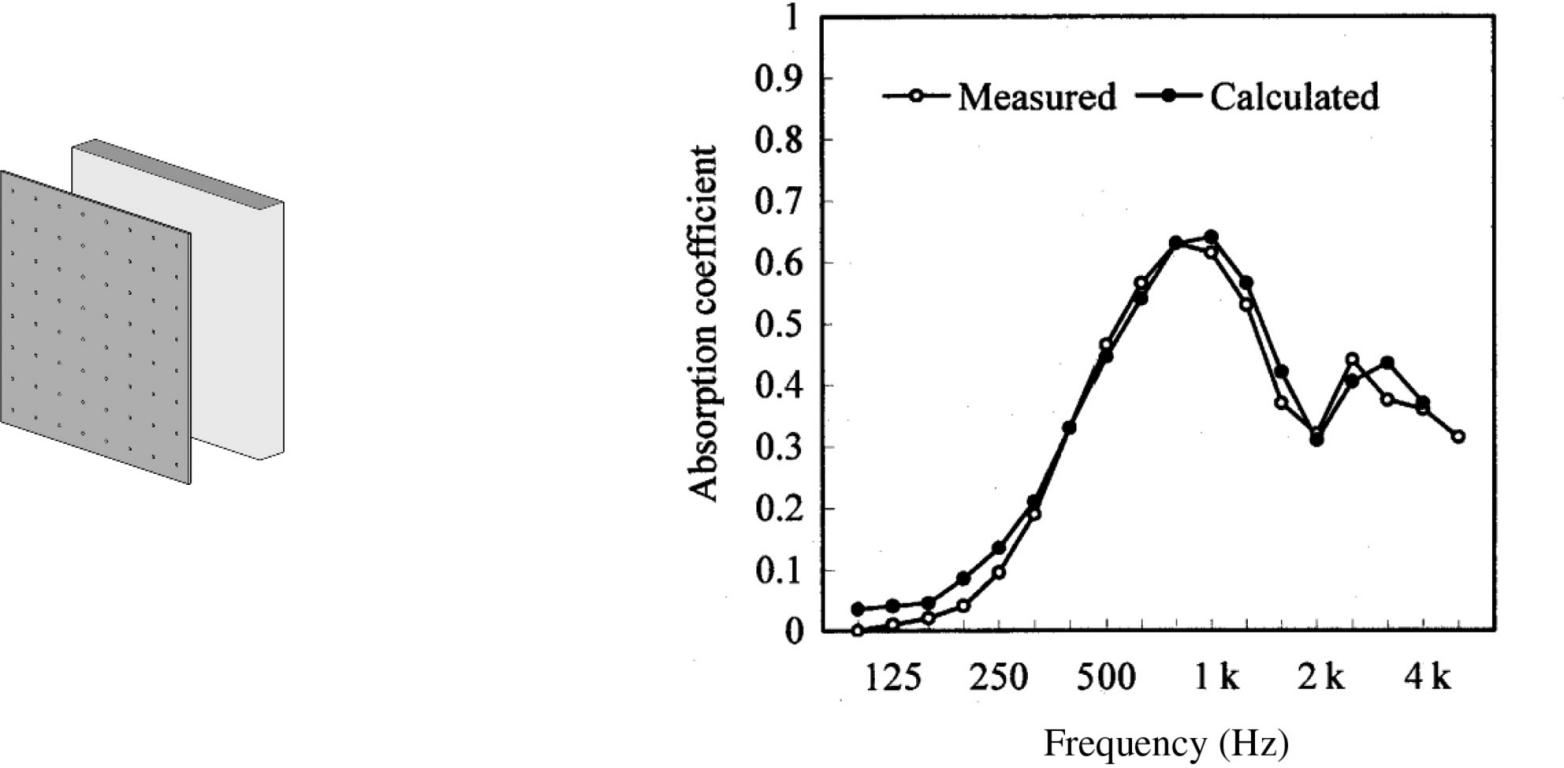
An example of a sketch of a conventional MPP absorber (left) and the diffuse-field sound absorption characteristics of a conventional MPP absorber (right). The theoretically calculated value and the measured value are compared. Hole diameter and thickness of the MPP are 0.5 mm, and the perforation ratio is 0.64%. The air-cavity depth between the MPP and the rigid back wall is 0.05 m. MPP = microperforated panel.

A microperforated panel (MPP) is one of the most promising of the so-called ‘next-generation sound absorbing materials’. Maa first produced an MPP in the 1970s and developed theories concerning their potential performance [11–13]. Since then, many studies on MPPs have been conducted [14,15]. MPP absorbers mainly show high sound absorption performance at medium to high frequencies, which are important for the speech transmission performance in architectural spaces [4]. Figure 2 shows a typical example of the sound absorption performance of a conventional MPP sound absorber with a rigid back wall. which shows a significant peak absorption in the mid-frequency range.

An MPP absorbs sound energy in the same way as a conventional perforation panel does, by forming a Helmholtz resonator, which needs a rigid backing and an air cavity behind it. In this sense, it is similar to conventional perforated panels. However, MPPs employ submillimetre holes to realise suitable acoustic resistance for high sound absorption performance. As mentioned, its conventional application involves placing it in front of a rigid back wall. As an MPP is usually thin (less than 1 mm), it lacks the strength required for interior walls in building spaces; therefore, it is commonly used in places where users cannot touch walls directly. Furthermore, once fixed to the wall as an interior finishing, it is difficult to be cleaned with disinfectant.

**Figure 3:**
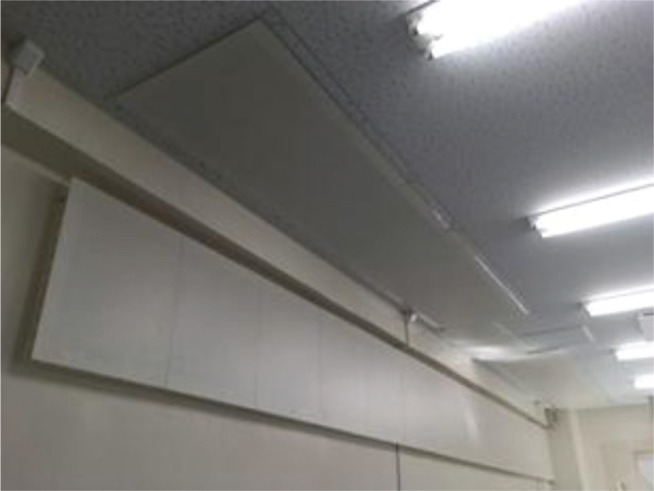
A photograph of a honeycomb-backed box-like MPP absorber panel installed on the corner of the wall and ceiling of a meeting room. The absorber panel is made of plastic sheets and is very lightweight. The surface of the MPP is painted to match the existing wall. These absorbers are suspended from a picture rail on the wall. MPP = microperforated panel.

As a trial implementation, Hoshi et al. [16] used a honeycomb-backed box-like MPP absorber panel, which was detachable from the main wall and could be suspended from the ceiling, etc., to improve excess reverberation and inferior speech intelligibility (see Fig. 3). This method is one possible solution applicable to ‘new style’ built environments.

**Figure 4:**
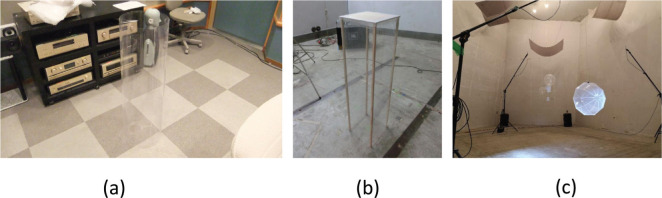
Photographs of the prototypes of (a) a CMSA, (b) an RMSA and (c) an SMSA. The CMSA and RMSA were made of polycarbonate MPP with a hole diameter and thickness of 0.5 mm and a perforation ratio of 0.785%. The SMSA was made out of a polypropylene sheet with the same parameters. CMSA = cylindrical MPP space absorber; MPP = microperforated panel; RMSA = rectangular MPP space absorber; SMSA = spherical MPP space absorber.

**Figure 5:**
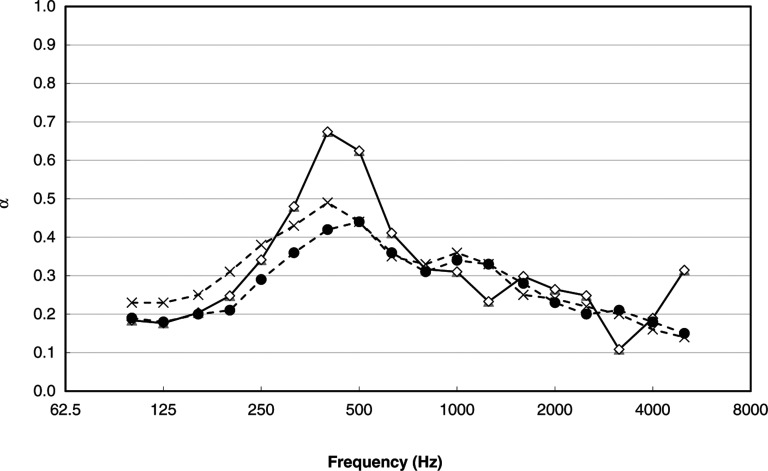
Measured diffuse-field equivalent sound absorption area for a 1-m^2^ surface area (i.e. equivalent to sound absorption coefficient). Crosses: CMSA; closed circles: RMSA; open circles: SMSA. As the characteristic dimensions of all specimens were almost the same, the peak frequency of the absorption due to the resonance was almost the same in the three types. CMSA = cylindrical microperforated panel space absorber; RMSA = rectangular MPP space absorber; SMSA = spherical MPP space absorber.

The concept of 3D-MPAs provides another alternative for the same purpose. This type of absorber can be used to improve the acoustics of an enclosed space without changing the design of its surfaces – it can simply be placed on the floor or suspended from the ceiling. Previously, the main variants of 3D-MPAs were the cylindrical shape [cylindrical MPP space absorber (CMSA)] [6], the rectangular shape [rectangular MPP space absorber (RMSA)] [7] and the spherical shape [spherical MPP space absorber (SMSA)] [8]. The prototypes of these 3D-MPAs and their sound absorptivity are shown in Figs 4 and 5.

CMSA and RMSA show a broad and mild peak at a resonance frequency. The peak value is c. 0.4–0.5, which is lower than conventional MPP absorbers with a rigid back wall. These 3D-MPAs show low additional absorption at low and high frequencies, which is a unique feature of 3D-MPAs and is not observed in conventional-type absorbers. Although the peak absorption is not high, they can be of some use in spaces where the original absorption is not enough. SMSAs show a rather sharp and high peak, with a value of c. 0.7. This may be more effective than a CMSA and an RMSA in some situations, for example, when a target frequency band is narrower. (See also Article Note 2.)

### Three-dimensional space sound absorbers with permeable membranes

PMs are traditional materials that have long been studied [17,18]. They absorb sound energy by their acoustic flow resistance; therefore, their absorption characteristics are similar to those of the porous/fibrous type. In traditional use, they are placed in front of a rigid back wall with an air-layer between the wall and the absorber, which is very similar to the application of porous/fibrous materials. In contrast to the frequency-selective absorption characteristics of MPPs, PMs show rather broad absorptivity, although limited to mid and high frequencies. Using a PM as a space sound absorber, a slight additional sound absorption is observed at low frequencies, which may be of some use in actual buildings as well [19]. A recent study of conventional PM absorbers with a rigid-back wall by Okuzono et al. [20] investigated the possibility of using various textiles (woven and non-woven) of different materials, which are recyclable. It was found that, as long as the flow resistance and areal mass are the same, the acoustic properties of PMs are not dependent on the material.

**Figure 6:**
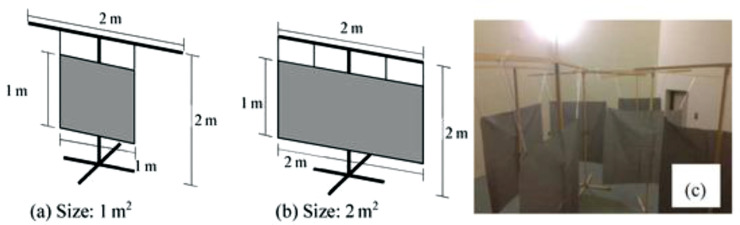
Overview of the studied specimens. (a) 1 m^2^ and (b) 2 m^2^ designs. (c) A photograph of specimens arranged in the reverberation chamber. The same configuration was applied to PMs of different flow resistance in the experiments. PM = permeable membrane.

**Figure 7:**
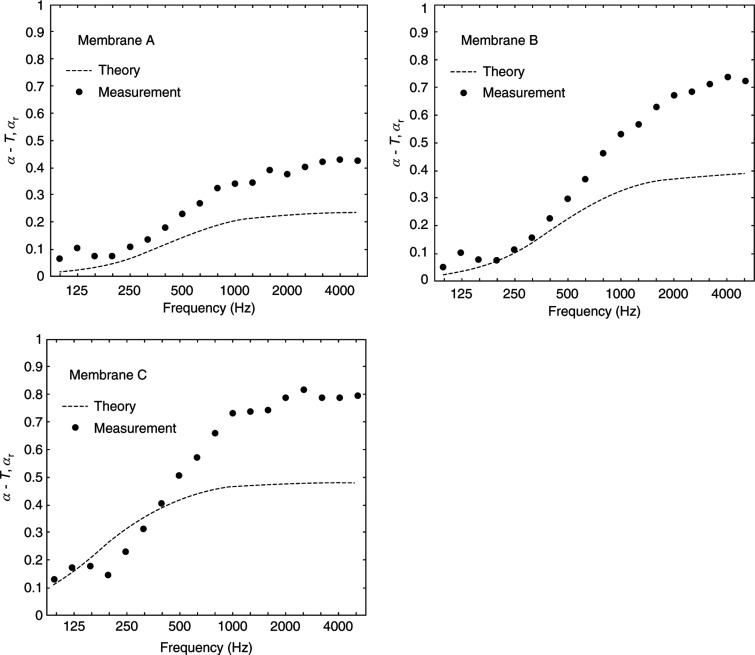
Measured diffuse-field absorptivity of rectangle shaped planar 3D-PMAs. Membranes A, B and C showed a flow resistance of 196, 462 and 1087 Pa s/m. The curves in each figure show the theoretical values, as presented in [9]. 3D-PMA = three-dimensional permeable membrane space sound absorber.

The simplest 3D space sound absorber with a PM (3D-PMA) is the rectangle plane 3D-PMA [9] (Fig. 6), which is very simple, as a rectangular PM is suspended from a frame or other structure. This is similar to an ‘acoustic curtain’, traditionally used in an auditorium to control the reverberation. Through the use of PMs, which are typically made of woven or non-woven textiles with polypropylene, polyester or polyethylene-terephthalate (PET), easily washable and durable absorbers which are suitable for ‘new style’ daily life can be made. The typical absorption characteristics of this type of absorber are shown in Fig. 7.

**Figure 8:**
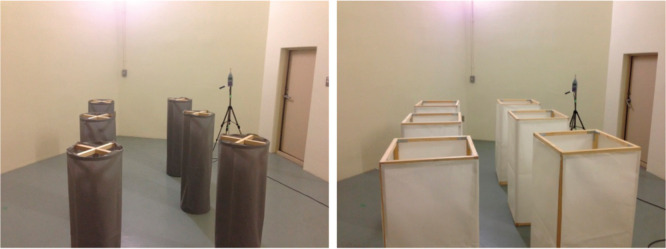
Prototype of 3D-PMA (left: cylindrical; right: rectangular) used in the experiment. 3D-PMA = three-dimensional permeable membrane space sound absorber.

In the case of PMs, other 3D shapes have been proposed, namely, cylindrical and rectangular [10] (Fig. 8). The difference between these and the 3D-MPA is that the 3D-PMA shows higher sound absorptivity in general if the flow resistance of the PM is properly selected, and wide-band sound absorptivity from mid to high frequencies. Therefore, if the absorption treatment is needed in a certain space, and it requires high sound absorptivity for one piece of absorber, the 3D-PMA may be a better choice. Furthermore, the flexibility of the 3D-PMA may also be advantageous in some cases. Due to the nature of the membrane, transparency cannot be realised by the PM, whereas it can be easily obtained in the case of MPPs.

**Figure 9:**
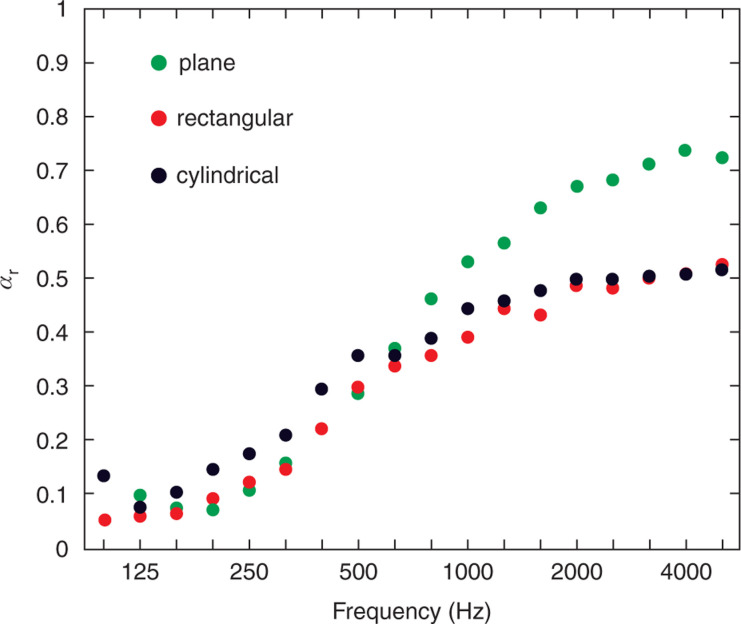
Comparison of the diffuse-field absorptivity of three types of 3D-PMA (planar rectangle, rectangular and cylindrical). The flow resistance of the membrane is 1087 Pa s/m and the surface area is 1 m^2^ in all cases. As observed, the planar rectangle 3D-PMA is the most efficient at high frequencies (>1000 Hz). At low frequencies, there is no significant difference, although the cylindrical type showed the highest values. 3D-PMA = three-dimensional permeable membrane space sound absorber.

Considering the shape of the 3D-PMA, as Fig. 9 clearly shows, the planar rectangle 3D-PMA is the most efficient at high frequencies. This is because of the area effect taking place along the edge of the membrane. If the mid to high frequencies are targeted, the planar rectangle type can be effectively used. This type is also the simplest and the easiest to prepare and apply to various situations. It would be a good choice for conditioning room acoustics without the need of for extra construction or refurbishing.

**Figure 10:**
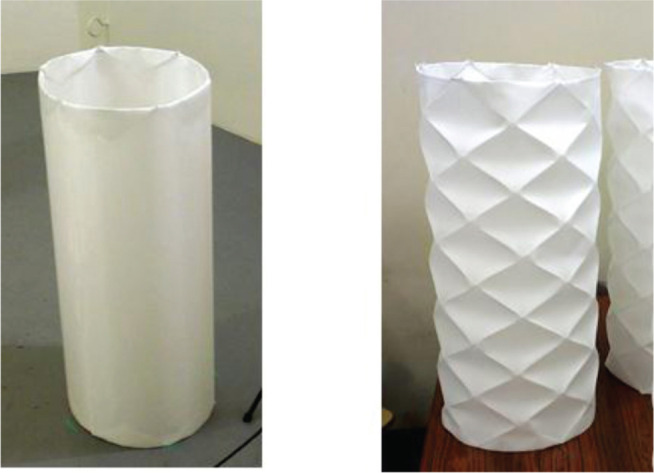
Prototypes of cylindrical 3D-PMA without surface unevenness (left) and with surface unevenness by means of the paper folding technique (right). 3D-PMA = three-dimensional permeable membrane space sound absorber.

Regarding the advantage of other shapes, for example, the cylindrical type can be arranged with surface roughness by means of the paper-folding technique, which may be appreciated for lighting purposes (details are provided in [21]). This type of absorber has an additional value as lighting equipment, as well as being a sound absorption tool (Fig. 10).

Recent developments in the study of 3D space sound absorbers include the theoretical study of the arbitrarily shaped 3D space sound absorbers [22]. With such developments, more variations of the shape of absorbers will possibly become available, which will give sound absorption technology the additional value of aesthetic designability to everyday use.

### Some simple examples

The effectiveness of sound absorbers depends on different conditions, for example, existing absorption, room volume, number of audience members, etc. Therefore, it is not easy to discuss the efficiency of those sound absorbers introduced already. The sound absorption performance can be evaluated by the sound absorption coefficient or the equivalent sound absorption area, or criteria derived therefrom. Therefore, some simple examples are presented here to demonstrate how the additional absorption may work. In the following example, we consider rather smaller rooms with typical existing equivalent absorption areas; however, it should be noted that the effect of additional absorption depends on the room size, existing absorption area, room shape and other factors. The purpose of the example is to show how additional absorption works in a simple way.

Considering that a typical value for the equivalent absorption area of an audience member is 0.37 per person (on average from 125 to 4 kHz), and noise reduction coefficient (NRC) is 0.39 [4], the absence of one person in the space may be compensated with a 3D-MPA with the equivalent sound absorption area of c. 0.4. Therefore, by using 3D-MPAs effectively, the lack of absorption can be improved by introducing them by a proper number in the case of a smaller space. However, when the space is large and the absence of a large number of audience members must be compensated for, this becomes more difficult, as many absorbers are required. In such cases, a 3D-PMA can be more efficient, as its equivalent sound absorption areas are higher than standard systems with comparable volume.

Another example can be given, considering the change in the reverberation time and adjusting it by additional absorptions through a simple calculation. Suppose that there is a multipurpose room of 10 × 15 × 3 m^3^ (volume: 450 m^3^, surface area: 600 m^2^ and audience seating area: 100 m^2^). It is assumed that the reverberation time of this room is 1.5 s, and that one person requires 1 m^2^ in the seating area. It is also assumed that the usual capacity of this room is 100 persons. The equivalent absorption area of the seating area can be supposed to be around 40 m^2^. If the number of audience members is reduced to half of the usual capacity, that is, 50 persons, the reverberation time can be 2.57 s. To adjust this to the original value, an additional absorption of 20 m^2^ is required. This may be relatively difficult, requiring nearly 30 space absorbers, each with an equivalent absorption area of 0.7 m^2^. However, by introducing this additional sound absorption, the acoustics of the room can be somewhat improved, even though some elaboration will be needed: for example, when these absorbers are used among the users of a room, they should be designed not to disturb the users. Such a consideration will need some more design consideration, such as a shape and colour, etc., based on ergonomics. Some elaboration will also be needed in adding additional function: for example, the sound-absorbing lampshade proposed in Ref. [21] has been improved to provide better luminescence distribution, however, it may need some more design consideration to optimise it as a lighting equipment according to rooms of different purposes.

## Concluding remarks

In this study, acoustical problems, which are likely or already taking place, namely, a lack of sound absorption, are discussed. This problem has existed before, but it is likely to increase in the post-pandemic period, as reduced numbers of audience members or users of a room become ‘standard’ in order to maintain social distance in enclosed spaces. Therefore, the lack of sound absorption in the rooms should be compensated for, by introducing additional sound absorbents.

Additional absorption treatments are in many cases very difficult to apply because they require considerable construction efforts and changes in architectural design. Therefore, to avoid this, one of the promising alternatives is the introduction of space sound absorbers.

Furthermore, in the ‘new lifestyle’ of the post-pandemic era, we must consider everything from the point of view of hygiene. Thus, the materials used should be washable and easily cleaned and sanitised. Considering these points, porous and fibrous materials such as wools or foams are not suitable. Therefore, sheets and panels such as MPPs and PMs are good choices.

In this study, we introduced and summarised the nature of 3D microperforated and PM space sound absorbers of various types, which resulted from our on-going project over several years. We presented their typical absorptive characteristics of the published results from our projects. As these absorbers have displayed not only practical absorption performance, but also a wide applicability and the potential value of aesthetic designability. By adding some considerations about ergonomics, lighting, and other purpose-oriented design considerations, we believe that these absorbers can offer an alternative solution to the acoustic challenges of the ‘new style’ built environment.

As shown, the sound absorbers are movable, so they can be rearranged as needed. In addition, they can be easily removed when they are no longer needed. Given the difficulty of predicting how long the ‘new style’ will last, it is considered that a method of controlling the sound field without major modifications to existing buildings will be needed. This can be a concept that will be required in some form for newly designed buildings as well. In any case, such a flexible and reversible design can be considered of some use, even if the ‘new style’ may only last a short while and our lifestyles return to the pre-pandemic ways.

## Data Availability

The datasets generated during and/or analysed during the current study are available from the corresponding author on reasonable request.

## Article note

(1) The guidelines for operating a performing art event in the ‘new style’ in Japan were revised in September 2020, and for some types of performing arts, venues can accommodate audiences up to 100% of the capacity. However, performers and performing spaces often reduce the number of audience members according to the former guidelines. (2) A detailed report of the 3D-MPA project is available in Kusaka et al. [23].
